# Which scale is more useful to detect diabetic neuropathic pain?: A cross-sectional study

**DOI:** 10.1186/s12902-022-00970-3

**Published:** 2022-03-07

**Authors:** Zeynep Ünlütürk, Saadet Nur Sena Öztekin, Hakan Alkan, Hande Şenol, Selin Betaş, Çağdaş Erdoğan

**Affiliations:** 1 Department of Neurology, Faculty of Medicine, Pamukkale University, Denizli, Turkey; 2 Giresun Prof. Dr. A. İlhan Özdemir State Hospital, Giresun, Turkey; 3 Department of Physical Therapy and Rehabilitation, Faculty of Medicine, Pamukkale University, Denizli, Turkey; 4 Department of Biostatistics, Faculty of Medicine, Pamukkale University, Denizli, Turkey

**Keywords:** Diabetic Neuropathy, Questionnaire, Neuropathic Pain, Diabetic Neuropathic Pain

## Abstract

**Background:**

Diabetic neuropathy is one of the most common causes of neuropathic pain. LANSS, sLANSS, DN4 and painDETECT are scales which are commonly used worldwide. There are not many studies comparing these screening tools in specific neuropathic pain subgroups.

The aim of this study is to compare the utilities of LANSS, sLANSS, DN4 and PainDETECT for the diagnosis of diabetic neuropathic pain.

**Methods:**

One hundred-one individuals without diabetic neuropathic pain were included in control group, 102 patients with diabetic neuropathic pain to DNP group. LANSS, sLANSS, DN4 and painDETECT scores of the groups were compared.

**Results:**

The difference between the groups was significant for all questionnaires and for all questions/titles they included. DN4 had the highest sensitivity and painDETECT had the highest specificity.

**Conclusions:**

All questionnaires seemed to be useful for detecting diabetic neuropathic pain. DN4 had a high specificity and sensitivity. PainDETECT, also had a high sensitivity and specificity when cut off value was accepted more than 12.

## Background

Neuropathic pain could be seen in up to 7–8% of the population [1] and one of the most common causes is diabetic neuropathy [2, 3]

Many screening tools were developed for the diagnosis of neuropathic pain but none of them have 100% diagnostic accuracy, so currently the gold standard for the diagnosis of neuropathic pain is still accepted to be the clinicians’ opinion [4].

Each screening tool has its own advantages and disadvantages. To use each scale is not possible in clinical practice or when grouping patients for clinical researches. The Leeds Assessment of Neuropathic Symptoms and Signs (LANSS), self Leeds Assessment of Neuropathic Symptoms and Signs (sLANSS), Douleur Neuropathique 4 (DN4) and painDETECT questionnaires are some of these scales which are commonly used worldwide and which have Turkish validation and reliability studies.

LANSS was developed in 2001 by Bennett [5] and Turkish version’s validity and reliability was demonstrated in 2004 [6]. It includes 5 symptom questioning and 2 examination parts. Bennet also modified the examination parts of LANSS in order to allow the patients to apply these stages to themselves, and developed sLANSS in 2005 [7] which also had a Turkish validation study [8].

Bouhassira developed DN4 in 2006, which had 10 items questioned under 4 titles including examination of allodynia and hyperalgesia [9]. Turkish validation study was performed in 2010 [10].

In 2006 Freynhagen developed painDETECT scale [11] which includes symptom questioning and also the type, severity and the radiation of pain. Turkish validation study was performed in 2013 [12].

The sensitivity and spesificity of these screening tools were reported to be 85% and 80% for LANSS, 74%—76% for sLANSS, 83%-90% for DN4 and 80%-85% for painDETECT in their original versions [5–12].

These screening tools were developed by studies using large study groups of neuropathic pain including many subgroups. There are not many studies designed for the utility of each screening tool in a specific neuropathic pain subgroup or comparing these screening tools in specific neuropathic pain subgroups. DN4 has a validation study for diabetic neuropathic pain but was not compared with other scales in that study [13].

The aim of this study is to compare the utilities of LANSS, sLANSS, DN4 and painDETECT for the diagnosis of diabetic neuropathic pain which is a common cause of neuropathic pain in clinical practice.

## Methods

The study was approved by the Clinical Research Ethics Committee of Pamukkale University (approval date: 21/11/2017 number: 77537). Informed consent was required from all participants included to the study.

102 patients who had submitted to outpatient clinic for neuropathic pain and were diagnosed as diabetic neuropathic pain by two different clinicians were included to the diabetic neuropathic pain (DNP) group. 101 patients who had submitted to Physical Rehabilitation outpatient clinic and whose pain was defined as non neuropathic pain by two clinicians were included to the control group.

All individuals were between 18–75 years old. The patients who were taking medicine that should be used for the treatment of neuropathic pain were excluded.

LANSS, sLANSS, DN4 and painDETECT questionnaires which had Turkish validity and reliability studies were applied to all individuals. Both groups scores were compared in order to demonstrate the utilities of these scales in the diagnosis and differential diagnosis of diabetic neuropathic pain. All 4 questionnaires were done by patients and clinician together, in same day and at one session. Physicians have done the questionnaires randomly.

The cut off values were accepted as the ones suggested in their original studies. For LANSS and sLANSS the scores ≥ 12 and for DN4 scores ≥ 4 were accepted as positive for the diagnosis of neuropathic pain. For painDETECT; scores ≤ 12 as negative, scores between 13 to 18 as unclear, and scores ≥ 19 as positive for the diagnosis of neuropathic pain.

Statistical Package for Social Science (IBM SPSS Statistics 25 software (Armonk, NY: IBM Corp.)) was used for data analysis. Continuous variables were denoted as mean ± standard deviation and categorical variables as numbers and percentages. Sensitivity, Specificity, Positive Predicted Value, Negative Predicted Value and ROC curves were used to examine method performances. *P* value above 0.05 was considered statistically significant.

## Results

Two hundred-three patients were included in the study. There were 102 patients, including 48 males and 53 females in the control group, and 44 males and 58 females in the diabetic neuropathic pain (DNP) group. There were not significant differences between the groups’ mean age and gender ratios (*p* > 0.05).

Mean painDETECT score was 4,7 ± 2,5 in control group and 19,8 ± 5,3 in the DNP group. The difference was significant (*p* = 0.00). Considering the cutoff values referred in its original version; scores between 0–12 were defined as negative, 13–18 were defined as unclear, and scores above 18 as positive for neuropathic pain. All 101 patients in the control group had scores smaller than 13(negative). Of the 102 patients in DNP group; 3 patients had negative, 47 patients had unclear and 52 patients had positive scores (Table 1).

**Table 1:** PainDETECT Questionnaire Results

PainDETECT Score		Control	Diabetic	Total
	Negative (0–12)	101 (%100)	3 (%2.9)	104 (%51.2)
	Unclear (13–18)	0 (%0)	47 (%46.1)	47 (%23.2)
	Positive (≥ 19)	0 (%0)	52 (%51)	52 (%25.6)
Total		101 (%49.8)	102 (%50.2)	203 (%100)

The difference between sLANSS scores of the groups was significant 2,91 ± 3,5for the control group and 15,4 ± 4,5 for DNP group (*p* = 0.000). As referred in their original versions for LANSS and sLANSS, scores lower than 12 were defined as negative and upper than 12 as positive for neuropathic pain. Ninety eight of 101 patients in the control group had negative and 3 had positive scores. Nineteen patients in DNP group had negative and 83 had positive values (Table 2).

**Table 2:** sLANSS Questionnaire Results

sLANSS Score		Control	Diabetic	Total
	Negative (0–11)	98 (97%)	19 (18.6%)	117 (57.6%)
	Positive (≥ 12)	3 (3%)	83 (81.4%)	86 (42.4%)
Total		101 (49.8%)	102 (50.2%)	203 (100%)

The mean LANSS score was 2,99 ± 4,1for the control group and 15,24 ± 4,3 for DNP group. The difference was significant (*p* = 0.000). Ninety seven of 101 patients in the control group had negative and 4 had positive scores. Twenty one patients in DNP group had negative and 81 had positive values (Table 3).

**Table 3:** LANSS Questionnaire Results

LANSS Score		Control	Diabetic	Total
	Negative (0–12)	97 (96%)	21 (20.6%)	118 (58.1%)
	Positive (≥ 12)	4 (4%)	81 (79.4%)	85 (41.9%)
Total		101 (49.8%)	102 (50.2%)	203 (100%)

The mean DN4 score was 1,13 ± 1,0the control group and 7,28 ± 1,5 in DNP group (*p* = 0.000). As referred in its original version scores lower than 4 were defined negative and 4 or more as positive. All patients in the control group and only 1 in DNP group had negative values (Table 4).

**Table 4:** DN4 Questionnaire Results

DN-4 Score		Control	Diabetic	Total
	Negative (0–4)	101 (100%)	1 (1%)	102 (50.2%)
	Positive (≥ 4)	0 (0%)	101 (99%)	101 (%49.8)
Total		101 (49.8%)	102 (50.2%)	203 (100%)

Every question of these 4 screening tools were compared one by one between all individuals of the groups. The difference was significant between groups for every question (*p* = 0.000).

The highest negative responses were to the items questioning hyperalgesia and allodynia in the control group, which were also not positive in all individuals of the DNP group (Table 5).

**Table 5:** The items questioning hyperalgesia and allodynia and answers by subgroups

Scale- Question		Control	Diabetic	*p* value
painDETECT—Allodynia (mean score ± SD)		0.2 ± 0.6	2.6 ± 1.6	0.000
painDETECT—Hyperalgesia (mean score ± SD)		0.6 ± 1.1	2.1 ± 1.6	0.000
LANSS—Allodynia	0 point (%)	95 (46.8%)	62 (30.5%)	0.000
	5 point (%)	6 (3%)	40 (19.7%)	
LANSS—Hyperalgesia	0 point (%)	93 (45.8%)	10 (4.9%)	0.000
	3 point (%)	8 (3.9%)	92 (45.4%)	
sLANSS—Allodynia	0 point (%)	101 (49.8%)	39 (19.2%)	0.00
	5 point (%)	0 (0%)	63 (31%)	
sLANSS—Hyperalgesia	0 point (%)	87 (42.9%)	33 (16.2%)	0.00
	3 point (%)	14 (6.9%)	69 (34%)	
DN4—Question 10	0 point (%)	98 (48.3%)	80 (39.4%)	0.00
	1 point (%)	3 (1.4%)	22 (10.9%)	

The title questioning skin color changes in LANSS and SLANSS is the most frequent one taking zero points in both control and even DNP groups( 87.1%—72.5% for LANSS and 87.1% -73.5% for sLANSS).

Correlation analysis demonstrated a significant, positive moderate correlations between the questionnaires used in this study for both groups (*r* value between 0.3–0.7). This correlation was more significant between LANSS and sLANSS (Table 6).

**Table 6:** Correlation analysis of scales (PD:PainDETECT, **: *r* value between 0.3–0.7; positive moderate correlations)

	*p*/*r* value		**PD**	**sLANSS**	**LANSS**	**DN4**
Control	PD	*r*	1,000	0,522^**^	0,470^**^	0,508^**^
		*p*		0,000	0,000	0,000
	sLANSS	*r*	0,522^**^	1,000	0,931^**^	0,485^**^
		*p*	0,000		0,000	0,000
	LANSS	*r*	0,470^**^	0,931^**^	1,000	0,486^**^
		*p*	0,000	0,000		0,000
	DN4	*r*	0,508^**^	0,485^**^	0,486^**^	1,000
		*p*	0,000	0,000	0,000	
Diabetic	PD	*r*	1,000	0,580^**^	0,486^**^	0,411^**^
		*p*		0,000	0,000	0,000
	sLANSS	*r*	0,580^**^	1,000	0,772^**^	0,413^**^
		*p*	0,000		0,000	0,000
	LANSS	*r*	0,486^**^	0,772^**^	1,000	0,356^**^
		*p*	0,000	0,000		0,000
	DN4	*r*	0,411^**^	0,413^**^	0,356^**^	1,000
		*p*	0,000	0,000	0,000	

**Fig. 1:**
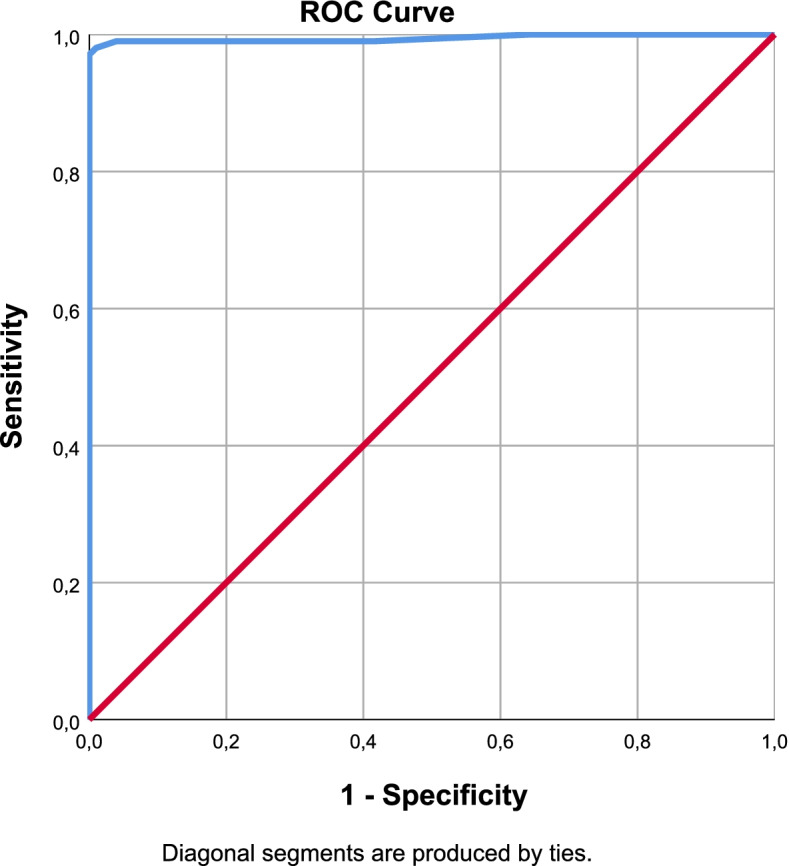
PainDETECT ROC Curve

**Fig. 2:**
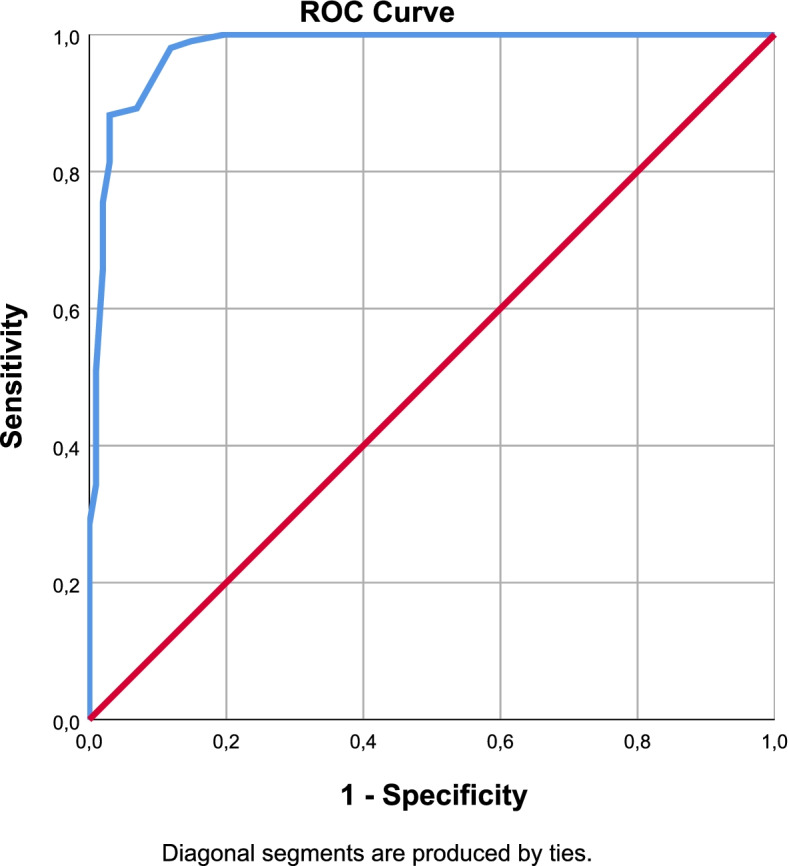
sLANSS ROC Curve

**Fig. 3:**
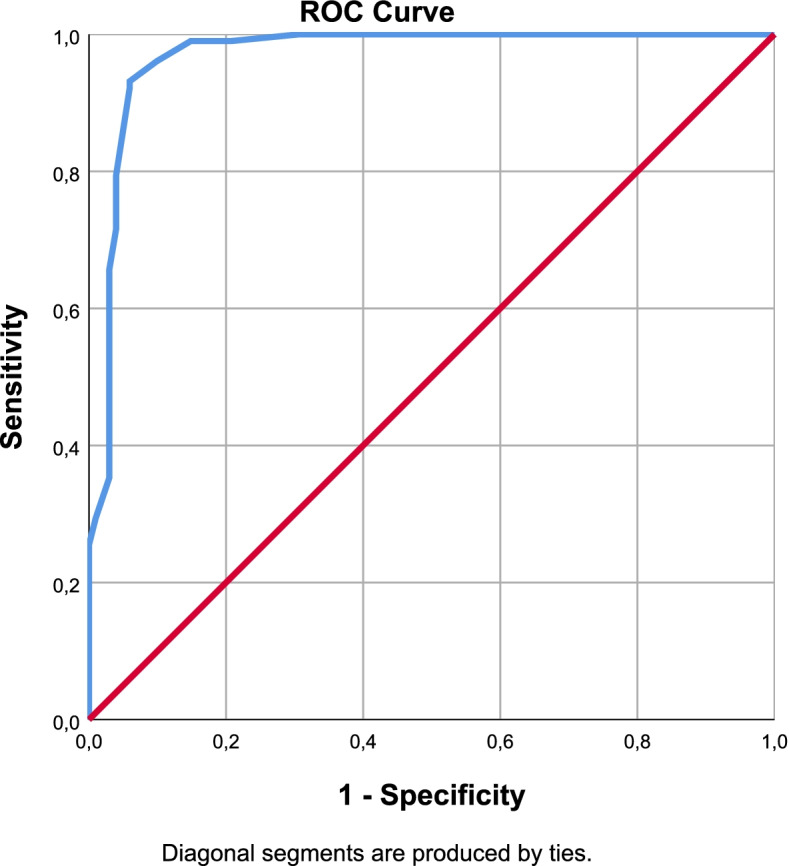
LANSS ROC Curve

**Fig. 4:**
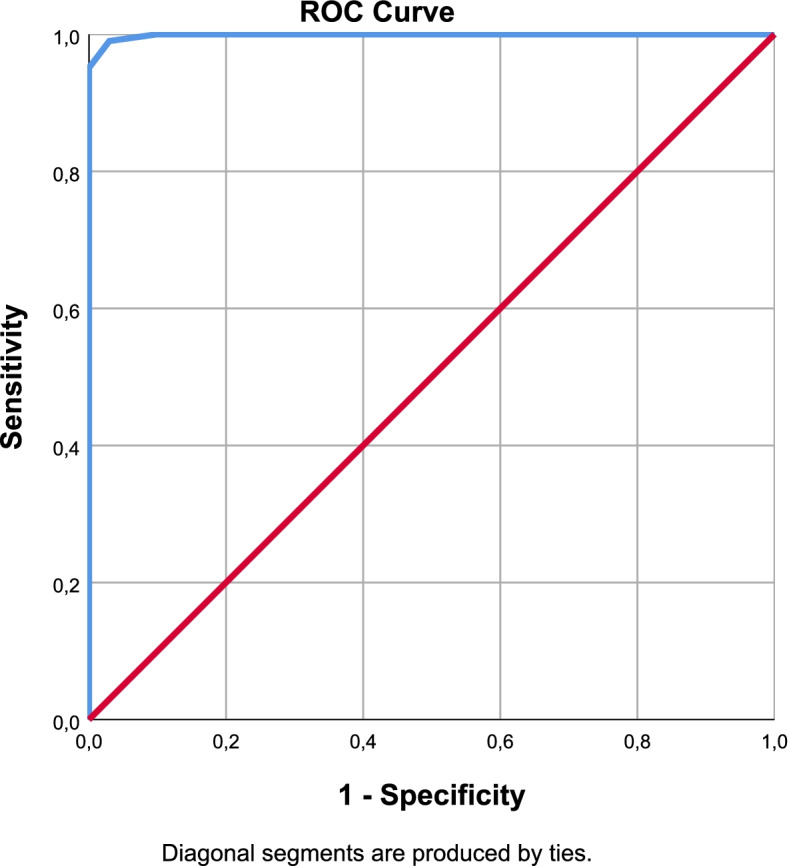
DN4 ROC Curve

To compare the performances of the scales that used in this study, we did Receiver Operating Characteristic (ROC) analysis. ROC Curves were made for each questionnaires (Figs. 1, 2, 3 and 4).

The cut-off values were defined by Youden analysis. As a result of the ROC analysis, Area Under the Curve (AUC), sensitivity and specificity values were interpreted for the diagnostic performance of the scales. With optimal cut-off points obtained from Youden Index; sensitivity, specificity, positive and negative predictive values were determined and performance results were examined. The optimal cut-off value for painDETECT was12.5 points with 97% sensitivity and 100% specificity. It was 9.5 points with 93% sensitivity and 94% specificity for LANSS and 7.5 points with 98% sensitivity, 88% specificity for sLANSS. For DN4 scale detected optimal cut-off value was 3.5 points with 99% sensitivity and 97% specificity. According to these cut-off values the diagnostic power of the questionnaires were evaluated as PD = DN4 > sLANSS > LANSS (Table 7).

**Table 7:** ROC Analysis of the Scales (AUC: Area Under the Curve S.E: Standart Error)

	AUC	S.E	p	Asymptotic 95% Confidence Interval			
				Lower Bound	Upper Bound	Youden Index	Optimal Cut Off
PainDETECT	0,999	0,001	0,000	0,998	1,000	0,971	12,5000
sLANSS	0,979	0,009	0,000	0,961	0,996	0,861	7,5000
LANSS	0,969	0,013	0,000	0,944	0,994	0,872	9,5000
DN4	0,999	0,001	0,000	0,997	1,000	0,960	3,5000

Patients with unclear results for painDETECT were either included in the negative and positive groups for neuropathic pain, and two separate sensitivity /specificity analyzes were performed and the findings are summarized in the table (Table 8). According to these results,

**Table 8:** Sensitivity, specificity of pain scales (ppv:positive predictive value, npv:negative predictive value)

Questionnaire	Sensitivity %	Specificity %	Ppv %	Npv %
PainDETECT (unclear = negative)	50,98	100	100	66.89
PainDETECT (unclear = positive)	97.06	100	100	97.12
sLANSS	81.37	97.03	96.51	83.76
LANSS	79.41	96.04	95.29	82.2
DN4	99.02	97.03	97.12	98.99

It was observed that the sensitivity of this scale decreased considerably among the unclear group was added to negative group in the painDETECT scale.

## Discussion

These questionnaires were developed for screening neuropathic pain conditions including diabetic neuropathic pain and many studies have demonstrated their utilities. As expected, the DNP groups’ scores were higher than control group for each screening tool.

These questionnaires were developed based on the fact that some symptoms are more expected in neuropathic pain than other pain conditions. The difference between the groups was significant for every title or question included in these questionnaires. Which supported that these symptoms are more frequent in neuropathic pain. It is also supported that all four questionnaires achieve the goal for selecting symptoms. But many symptoms were also detected in the control group which once more underlines the fact that these symptoms are more frequent in neuropathic pain but not specific to neuropathic pain.

The differences between the groups were more significant for the titles allodynia and hyperalgesia which are evoked symptoms requiring examination. This suggests that physical examination contributes the diagnostic process in neuropathic pain as it is in many condition. So the screening tools containing examination titles may have an advantage for the diagnostic accuracy. Others without these titles may have the advantage to be easier to use or to take less time.

Another point taking attention is that the title taking more zero points in both groups was the skin color changes questioned in LANSS and sLANSS. This title was scored zero to five points in these scales. And was negative for 73.5–72.5% in even the DNP group. Also hyperalgesia and allodynia were screened in both questioning and examination parts of LANSS and sLANSS. As it is in clinical practice, our results also suggested that allodynia and hyperalgesia may not be present in every patient with diabetic neuropathic pain. So in the absence of skin color changes, hyperalgesia and allodynia, a patient total score directly decreases from 24 to 13 points, which could cause difficulty when screening neuropathic pain even it exists.

Our results demonstrated a significant positive correlation between the screening tools suggesting that each questionnaire supports the other ones results. These are all worldwide accepted screening tools and their utility were tested in many studies [14]. So similar results should be expected. The correlation was strongest between LANSS and its modified form sLANSS which also was expected.

Though these screening tools are used in many studies for many different neuropathic pain subgroups, there are not so many studies comparing these scales in a distinct subgroup.

A study comparing DN4, PainDETECT, LANSS, Identification Pain (ID-Pain) and Neuropathic Pain Questionnaire (NPQ) for detecting neuropathic pain reported that DN4 and NPQ should be the most useful ones for clinical purpose; but also mentioned, that they may not get ahead of clinicians’ opinion [15].

Another study comparing DN4, painDETECT, NPQ and LANSS in chronic pain suggested that DN4 might be the more sensitive and LANSS might be the less [16].

A comparison of DN4, painDETECT, NPQ and LANSS in spinal cord injury reported a diagnostic accuracy of 88% for DN4, 78% for PainDETECT, 65% for NPQ and 55% for LANSS [17].

A study for validation of DN4 in painful diabetic neuropathy reported that the sensitivity was 80% and the specificity was 92% for the cutoff value 4 [13].

Another study designed to evaluate the validity and reliability of LANSS in Libya, included diabetic patients to the study and demonstrated that it was useful [18].

A study comparing LANSS and DN4 in diabetic neuropathic pain in China reported that the sensitivity was 82.7% for DN4 and 58% for LANSS. The specificity was 97% for both [19].

In this study we aimed to compare the utilities of 4 screening tools; LANSS, sLANSS, DN4 and painDETECT for detecting diabetic neuropathic pain, which to our knowledge was not present in the literature. We thought it would give additional information about their usage in clinical practice.

Our results suggested that DN4 had high specificity and sensitivity for screening diabetic neuropathic pain which supported the data reported in previous studies [13, 19].

Similar to previous studies, our data also suggested that LANSS and sLANSS should also be used for screening diabetic neuropathic pain but seems to have a lower sensitivity and specificity [16, 17, 19].

Among these screening tools, there seems to be various results for painDETECT in the literature which might be caused by the different cutoff values this scale includes. In our study if the cutoff value was accepted as 19 points, the sensitivity decreased. If the cutoff value was accepted as 13 the sensitivity was high as DN4 (97%). The specificity was 100% for each cutoff values. The observation that there were no patients with more than 12 points in the control group suggested that cutoff value of 13 should be useful when using painDETECT for screening diabetic neuropathic pain. Additional clinician observation should be needed for the individuals who had 13 to 18 points.

Another fact about these screening tools for detecting neuropathic pain is that DN4 and LANSS include physical examination while painDETECT and sLANSS do not.

In the light of foregoing, we would like to state once again that the gold standard of the diagnosis diabetic neuropathic pain is the clinician's opinion. However, DN4 seems to be one step ahead of the other scales evaluated in this study in terms of not requiring two separate cut-off values and being easy and practical to use in daily clinical routine. The percentages calculated in our study should not be considered as alternative values for the ones demonstrated in large population studies designed to detect neuropathic pain. Also to talk about their specificities and sensitivities in subgroups like diabetic neuropathic pain, there should be more studies including larger patient populations.

## Conclusions

All screening tools seemed to be useful for detecting diabetic neuropathic pain and correlated each other. DN4 and painDETECT (when cut off value was 13) seemed to have higher sensitivity and specificity.

## Data Availability

The datasets used and/or analyzed during the current study are available from the corresponding author on reasonable request.

## Abbreviations

LANSS: The Leeds Assessment of Neuropathic Symptoms and Signs
sLANSS: Self Leeds Assessment of Neuropathic Symptoms and Signs
DN4: Douleur Neuropathique 4
PD: PainDETECT
DNP: Diabetic neuropathic pain
AUC: Area Under the Curve
S.E: Standart Error
PPV: Positive Predictive Value
NPV: Negative Predictive Value
ID Pain: Identification Pain
NPQ: Neuropathic Pain Questionnaire
